# Clinical features and outcome of sporadic serogroup W135 disease Taiwan

**DOI:** 10.1186/1471-2334-6-7

**Published:** 2006-01-19

**Authors:** Jiun-Ling Wang, Ding-Ping Liu, Jer-Jea Yen, Chia-Jung Yu, Hsi-Chen Liu, Ching-Yuang Lin, Shan-Chwen Chang

**Affiliations:** 1 Department of Internal Medicine, National Taiwan University Hospital and National Taiwan University College of Medicine, Taipei, Taiwan; 2 Division of Immunization, Center for Disease Control, Department of Health, Taiwan; 3 Institute of Medical Research, College of Health Sciences, Chang Jung Christian University, Taiwan

## Abstract

**Background:**

Few published reports have evaluated the clinical features and outcome of serogroup W135 meningococcal disease. In Taiwan, W135 is the second most prevalent meningococcal disease serogroup.

**Method:**

A nationwide study was conducted to retrospectively analyze epidemiologic data from 115 patients with laboratory confirmed meningococcal disease that occurred from 2001 through 2003.

**Results:**

Serogroup W135 accounted for 26% of all cases and most (76.7%) were older than 20 years. There were no cases of serogroup W135 meningococcal disease associated with Hajj pilgrims, and all cases were sporadic. In 88 patients with complete case records, we compared the presenting symptoms, signs, laboratory data, and outcomes between W135 and non-W135 patients. There were no differences in presenting symptoms except for the higher prevalence of pneumonia found in W135 patients (23.8% *vs. *1.5%; OR: 20.6; 95%CI: 2.3–189.0; p = 0.003). The distribution of inflammatory cells in CSF in patients with meningitis was also different between W135 and non-W135 patients. W135 patients had a trend toward more intubations and shock but it did not achieve statistical significance. In multivariate analysis of factors associated with death, three independent factors were found: bacteremia without meningitis, altered mental status, and petechiae or purpura on admission.

**Conclusion:**

Sporadic serogroup W135 meningococcal disease is an important component of the meningococcal disease burden in Taiwan, but it is not directly associated with Hajj pilgrims. Compared with patients infected by other serogroups of meningococci, patients with serogroup W135 were older and more likely to have extrameningeal involvement such as pneumonia.

## Background

Previous surveys of meningococcal disease in the United States and elsewhere found serogroup W135 was rare [1]. An outbreak of serogroup W135 meningococcal infection in Saudi Arabia following the 2000 Hajj was reported, and then spread to several countries around the world [2-4]. Significantly higher carriage rate of *N. meningitidis *serogroup W135 in pilgrims returning from the Hajj [5,6] and continuing diversification of the serogroup after its emergence in 2000 has been found [7,8]. In Taiwan, only about 2% (50,000) of the population are Muslims. In the period from 2001 through 2003, about 20 to 40 pilgrims per year went from Taiwan to Saudi Arabia for the Hajj (data from Chinese Muslim Association, Taiwan). Little is known about the clinical features and outcome of serogroup W135 disease [9-11], but there are some reports of extrameningeal complications [11-13].

The incidence of meningococcal disease in Taiwan was below 0.001 from 1980 to 1987, and re-emerged in 2000 with a rate of 0.07/10^5 ^population. In 2001 there was a further increase in incidence (0.19/10^5^) [14]. Serogroup B was the most common and W135 was secondmost predominant, which is different from other country [1,14]. To determine the clinical characteristics and outcome of patients with serogroup W135 and non-W135 meningococcal disease in Taiwan, a nationwide study was conducted from January 1, 2001, through December 31, 2003. The relationship between *N. meningitidis *serogroups with respect to patient characteristics, clinical manifestations, and outcome was assessed. The factors associated with mortality in meningococcal disease were also investigated.

## Methods

### Case reporting and microbiology laboratory procedures

In the National Notifiable Disease Surveillance System in Taiwan, patients with sudden onset of fever, headache, nausea, vomiting, stiff neck, petechial rash with pink macules, accompanied by delirium, coma or shock; or Gram-negative diplococci were found in smear of cerebrospinal fluid by Gram-stain should be reported to the Center for Disease Control (CDC), Taiwan as suspected cases of invasive meningococcal disease within 24 hours. In addition to the routine examination and culture in individual hospital, blood and/or cerebrospinal fluid of the suspect *N. meningitidis *would be sent to the bacteriology laboratory of Taiwan CDC at room temperature as soon as possible for bacteria culture and serogrouping. If *N. meningitidis *is isolated and the patient had compatible clinical symptoms and signs, the patient would be justified as a confirmed case of invasive meningococcal disease. If *N. meningitidis *is isolated in bacteriology laboratory of individual hospital, the isolate would be sent to Taiwan CDC laboratory. The identification of all isolates were reconfirmed at Taiwan CDC using conventional biochemical methods [15]. Serogrouping using the agglutination test (Murex Biotech Ltd, Dartford, UK) and standard grouping sera for capsular types A, B, C, X, Y, Z, and W-135 was also performed at Taiwan CDC.

### Epidemiological investigation

Within 48 hours since suspected case reported, the staffs at local Health Bureau will conduct the case investigation, to understand the detail travel history and identify every single close contact during the 2 weeks before disease onset. Patients or their family who have traveled abroad 3 months before onset were recorded. If any close contact develops fever, the person would be send to hospital immediately for examination, sampling and treatment. The close contacts with no related symptoms would receive prophylaxis as soon as possible.

### Clinical information collection

From January 2001 through December 2003, case-record forms designed for collection of detailed clinical data were send to and filled out by those physicians who reported the laboratory-confirmed cases. The information provided by the case-record forms included patients' history, symptoms and signs on admission, laboratory findings on admission, clinical course, treatment, outcome, and neurological findings at discharge. Patients were categorized as having either meningitis or pure bacteremia (the latter was defined as pure bacteremia if patients had neither meningitis nor abnormal cerebrospinal fluid analysis). Bacteremia without sepsis was defined as growth of *N. meningitidis *on blood agar in patients with fever but no signs of meningococcemia or meningitis [16]. Pneumonia was defined if consolidation or pleural effusion was seen on chest radiograph. Arthritis was present if the classic inflammatory signs of redness, swelling, heat and pain in the joint(s) were found.

### Statistics

The association of the following parameters (serogroup, age, sex, clinical category, initial symptoms and signs, and hemogram value) with mortality was analyzed. Statistical analyses were performed using SPSS software, version 10.0 (SPSS, Inc., Chicago, Ill, USA). The Chi-square test was used to assess the statistical significance of difference in the epidemiologic data, clinical manifestations, and outcome between serogroup W135 and non-W135 cases. Logistic regression was used to find the factors associated with attributable death.

## Results

### Patient characteristics and risk factors

From January 2001 through December 2003, there were 115 cases of laboratory confirmed invasive meningococcal disease reported to Taiwan CDC. The most common serogroup was serogroup B (47.8 %, n = 55), followed by serogroup W135 (26.1%, n = 30), serogroup Y (14.8 %, n = 17), nongroupable (5.2%, n = 6), serogroup C (3.5%, n = 4), and serogroup A (2.6 %, n = 3). The differences in age, sex distribution, living area, travel history, and case fatality rate between those with serogroup W135 and non-W135 meningococcal infections are shown in Table 1. Compared with patients infected by other serogroups of meningococci, patients with serogroup W135 were older. The median age of patients with serogroup W135, Y, others and B were 22, 19, 11, and 8, respectively. Serogroup W135 occurred more frequently in older patients (i.e., 20–54 years; 50.0% *vs. *25.9%, *P *= 0.015), while non-W135 serogroup infections occurred more commonly in patients younger than 10 years (83.3% *vs. *55.2%, *P *= 0.005). In adult patients older than 16 years old, the percentage of immunocompromised patients (cancer, steroid use, and organ failure) in serogroup W135 was not different from non-W135 group. There was no difference in the history of traveling abroad of patients or their household contacts with serogroup W135 or non-W135 (*P *> 0.05). No patients or their household contacts had traveled to Mecca and Medina in Saudi Arabia for the Hajj. No epidemic outbreaks or case clusters were reported during the study period, except for two patients with serogroup B who were from one family and had definitely been exposed to each other.

**Table 1:** Epidemiological data of patients with serogroup W135 and Non-W135 meningococcal disease.

Parameter	W135 N = 30	Non-W135 N = 85	Total N = 115	*P*
Male	17(56.7)	44(51.8)	61(53.0)	0.644
Age distribution				0.030
<1 year	5(16.7)	22(25.9)	27(23.5)	0.306
1–9 years	0(0)	16(18.8)	16(13.9)	0.011
10–19 years	5(16.7)	11(12.9)	16(13.9)	0.759
20–54 years	15 (50.0)	22(25.9)	37(32.2)	0.015
≧55 years	5(16.7)	14(16.5)	19(16.5)	0.980
College & high school students	1(3.3)	5(5.9)	6(5.2)	1.000
Military personnel	5(16.7)	11(12.9)	16(13.9)	0.759
Living area				0.385
North Taiwan	14(46.7)	43(50.6)	57(49.6)	
Central Taiwan	5(17.2)	21(24.4)	26(22.6)	
South Taiwan	10(34.5)	16(18.6)	26(22.6)	
East Taiwan	1(3.4)	5(5.8)	6(5.2)	
With travel abroad history ^a^	1(3.3)	8(9.4)	9(7.8)	0.442
Deaths	6(20.0)	22(25.9)	28(24.3)	0.519

NOTE. Data are no. (%) of patients, unless otherwise indicated.

^a ^Any patients and/or their family with travel abroad history 3 months before disease were recorded.

Compared with patients infected by serogroup B meningococci, patients with non-serogroup B were older (median age: non-serogroup B vs. serogroup B: 20 vs. 8 years old). Non-serogroup B patients occurred more in the age group "10–19" and "20–54" years (*P *< 0.05), while serogroup B infections occurred more commonly in the age group younger than 10 years (52.7% vs. 23.3%, *P *= 0.002). There were more military personnel in non-serogroup B than serogroup B (20.0% vs. 7.3%, *P *= 0.049).

### Comparison clinical features between patients with serogroup W135 and non-W135

Complete detailed clinical information was available on 77% (88/115) of patients with confirmed meningococcal disease. The age and serogroup distribution were not different between groups having and lacking complete information. The frequency of presenting symptoms, signs, and laboratory findings of the 88 patients are shown in Table 2. These 88 included 41 with serogroup B, 21 with W135, 15 with Y, 3 with C, 2 with A, and 6 with nongroupable meningococci. Fifty (56.8%) out of 88 cases had meningitis, and 38 (43.2%) had pure bacteremia without meningitis, including 7 cases with bacteremia without sepsis, 6 with pneumonia, and 3 with arthritis. Patients with serogroup W135 disease tended to have pure bacteremia without meningitis (52.4% *vs. *40.3%; OR: 1.6; 95%CI: 0.6–4.4) and less meningitis than the non-W135 patients, but the difference did not achieve statistical significance. Patients infected with serogroup W135 were more likely to have pneumonia (23.8% *vs. *1.5%; OR: 20.6; 95%CI: 2.3–189.0; p = 0.003). The initial presenting symptoms, signs (Table 2), interval between onset of symptoms and the first visit to the doctor, and interval between initial presentation and treatment with the first antibiotic did not differ between W135 and non-W135 patients. Subgroup analysis of meningitis patients showed those with serogroup W135 were less likely to have cerebrospinal fluid white cell count higher than 1000/ml. Of the 50 patients with meningitis, 62% (31) had positive blood culture.

**Table 2:** Clinical manifestations, laboratory data, and outcome of 88 meningococcal disease patients with completed case records: serogroup W135 *versus *non-W135.

Feature	W135 (n = 21)	Non-W135 (n = 67)	Total (n = 88)	P
No underlying disease	16(76.2)	53(79.1)	69(78.4)	0.768
Underlying disease of cancer	2(9.5)	4(6.0)	6(6.8)	0.626
Steroid use	2(9.5)	2(3.0)	4(4.5)	0.240
Disease classification				
Meningitis	10(47.6)	40(59.7)	50(56.8)	0.329
Bacteremia without meningitis	11(52.4)	27(40.3)	38(43.2)	0.329
Bacteremia without sepsis	2(9.5)	5(7.5)	7(8.0)	0.670
With pneumonia	5(23.8)	1(1.5)	6(6.8)	0.003
With arthritis	1(4.8)	2(3.0)	3(3.4)	0.563
Presentation				
Presentation ≤1 day after symptom onset	12 (57.1)	45 (67.2)	57 (64.8)	0.402
Presentation days (mean ± SD)	1.9 ± 2.4	1.7 ± 3.0	1.8 ± 2.9	0.810
Fever	17(81.0)	58(86.6)	75(85.2)	0.501
Sore throat	6(28.6)	16(23.9)	22(25.0)	0.665
Cough	12(57.1)	29(43.3)	41(46.6)	0.267
Myalgia and/or arthragia	5(23.8)	18(26.9)	23(26.1)	0.781
Nausea and/or vomiting	8(38.1)	35(52.2)	43(48.9)	0.258
Headache	7(33.3)	26(38.8)	33(37.5)	0.651
Petechia and/or purpura	6(28.6)	26(38.8)	32(36.4)	0.395
Altered mental status	8(38.1)	31(46.3)	39(44.3)	0.511
Seizures	2(9.5)	5(7.5)	7(8.0)	0.670
Laboratory data				
WBC count < 10,000/mm^3^	10(47.6)	26(38.8)	36(40.9)	0.474
Platelet < 100,000/mm^3^	6(28.6)	22(32.8)	28(31.8)	0.714
Cerebrospinal fluid^a^				
WBC count >1000/mm^3^	3/10(30.0)	29/40(72.5)	32/50(64.0)	0.024
WBC count 100–1000/mm^3^	5/10(50.0)	8/40(20.0)	13/50(26.0)	0.101
WBC count <100/mm^3^	2/10(20.0)	3/40(7.5)	5/50(10.0)	0.258
Protein > 300 mg/dl	4/9(44.4)	11/36(30.6)	15/45(33.3)	0.454
Glucose < 10 mg/dl	5/9(55.6)	18/35(51.4)	23/44(52.3)	1.000
Days from presentation to antibiotics use (mean ± SD)	0.7 ± 1.0	2.2 ± 5.0	1.8 ± 4.4	0.200
Outcome				
Shock	11(52.4)	21(31.3)	32 (36.4)	0.080
Intubation	9 (42.9)	16(23.9)	25(28.4)	0.092
Attributable death	4(19.0)	15(22.4)	19(21.6)	1.000
Death within 48 hours	2(9.5)	13(19.4)	15(17.0)	0.293

NOTE. Data are no. (%) of patients, unless otherwise indicated. WBC: white blood cells.

^a ^Cerebrospinal fluid data were analyzed in meningitis patients only.

### Outcome analysis

In outcome analysis, W135 patients tended to have more intubations (42.9% *vs. *23.9; OR: 2.4; 95%CI: 0.9–6.7; p = 0.09) and shock (52.4% *vs. *31.3%; OR: 2.4; 95%CI: 0.9–6.5; p = 0.08), but the difference did not reach statistical significance.

Twenty-two percent (19/88) of the patients died and their deaths were attributed to meningococcal infection. Fifteen (79%) of these occurred within the first 48 hours of presentation. By univariate analysis, the following factors were associated with death attributed to meningococcal disease: pure bacteremia without meningitis, altered mental status, WBC count of < 10,000/mm^3^, thrombocytopenia (platelet < 100,000/mm^3^), initial systolic blood pressure < 85 mmHg, and purpura or petechia at time of presentation to the hospital (p < 0.05) (Table 3). Using a multivariate regression analysis model, after adjusting for age, pure bacteremia (OR: 76.7, P < 0.001), altered mental status (OR: 44.2, p < 0.001), and petechia or purpura at presentation (OR: 6.4, P = 0.031) were associated with increased risk of mortality (Table 3). Using either a univariate or multivariate analysis model, serogroup was not found to be a significant risk factor for mortality.

**Table 3:** Significant factors associated with attributable death by univariate and multivariate analysis.

Factors	Univariate OR (95% CI)	*P*	Multivariate* OR (95% CI)	*P*
Age > 16	0.4(0.1–1.1)	0.081		
Male Sex	2.6(0.9–7.6)	0.067		
Pure bacteremia	11.4(3.0–43.2)	<0.001	76.7(8.8–672.0)	<0.001
Serogroup W135	0.8(0.2–2.8)	0.746		
Serogroup B	0.8(0.3–2.2)	0.658		
Steroid use	3.9(0.5–30.0)	0.186		
Malignancy	0.7(0.1–6.5)	0.762		
Any underlying disease	0.7(0.2–2.3)	0.573		
Fever>40°C	0.4(0.1–3.6)	0.433		
Hypothermia < 36°C	3.8(1.0–14.0)	0.050		
Diarrhea	1.8(0.4–9.0)	0.474		
Nausea or vomiting	1.1(0.4–3.0)	0.883		
Myalgia or arthragia	0.7(0.2–2.2)	0.543		
Altered mental status	7.0(2.1–23.6)	0.001	44.2(5.7–340.8)	<0.001
Purpura or petechia	4.2(1.4–12.2)	0.008	6.4(1.2–34.8)	0.031
WBC < 10,000/mm^3^	6.0(1.9–18.7)	0.002		
Platelet < 100,000/mm^3^	5.7(1.9–16.8)	0.002		
Systolic BP < 85 mmHg	5.2(1.5–17.4)	0.008		

*Logistic regression after adjusting for age (forward Wald method).

## Discussion

This nationwide study of meningococcal disease in Taiwan focused on the sporadic occurrence of serogroup W135 meningococcal disease. W135 serogroup accounted for 26% of all meningococcal disease in Taiwan, and its prevalence is second only to that of serogroup B. During the 1990s, *N. meningitidis *W135 represented less than 5% of all reported *N. meningitidis *in the UK, France, and the United States (1, 17–18). Taiwan is not a Muslim country and less than 40 persons per year are Hajj pilgrims from Taiwan. No case had contact with Hajj pilgrims in this series and analysis of the geographic distribution and timing of our serogroup W135 meningococcal disease cases did not suggest an epidemiologic link to Hajj pilgrims. The nasopharyngeal carrier rate of *N. meningitidis *was 2.3% in a nationwide surveillance from military recruits in Taiwan in 2001, and serogroup W135 constituted 15.2% [Taiwan CDC, unpublished data].

From pulsed-field gel electrophoresis and multilocus sequence typing genotyping in Taiwan CDC, serogroup B isolates in Taiwan were derived from several distinct lineages, but isolates of serogroup A, serogroups W135 and C, and serogroup Y, respectively, belonged to ST-7, ST-11, and ST-23 clones [19]. All our serogroup W135 meningococcal strains in Taiwan available for analysis in 2001–2002 belonged to ST-11/ electrophoretic type-37 complex, and they were apparently introduced before the Hajj pilgrimage outbreak [19]. And multilocus variable-number tandem repeat analysis with better genotyping discrimination power also showed that there were no a major epidemic *N. meningitidis *strain circulating in the country [Chiou CS, et el, unpublished data]. Our basic epidemiological data and the results of genotyping method by Chiou CS et al do not suggest that the 2000-Hajj epidemic directly lead to the sporadic cases of serogroup W135 meningococcal disease in Taiwan.

Few published papers discuss the clinical characteristics of serogroup W135 meningococcal disease, and all focus on cases caused by the epidemic strain [3,4]. But these studies [3,4] did not report finding more W135 than non-W135 patients who were older and in the 20–54 year-old age group. In case series from Europe and Saudi Arabia, patients 1–10 years-old accounted for nearly 50% of all cases of serogroup W135 meningococcal disease [4,20]. The cause of shift in the age distribution for W135 cases in our series was not clear. Meningococcal disease due to uncommon serogroups such as X, Y, Z, W135, has been reported associated with complement deficiency [21]. In our meningococcal patients older than 16 years old with complement 50 level and C3a level available (31/62,50%), serogroup W135 did not have more patients with low complement 50 and C3a level than serogroup B (28.6%; 4/14 vs. 47.1%; 8/17)(Lin CY, unpublished data). In adult patients elder than 16 years old, only 4 patients (28.6%) of serogroup W135 meningococcal disease had an underlying immunocompromising disease (cancer, steroid use, and organ failure etc) and were not different from non-W135 group. Our study showed that 83.3% of serogroup W135 disease and 76.7% of non-serogroup B occurred in patients older than 10 years – an age group in which the disease is potentially preventable by use of the current quadrivalent vaccine. In Taiwan, the current policy is to recommend meningococcal vaccination for people travelling to an endemic country, or for use in controlling outbreaks in crowded settings such as a school or military establishment.

If continued bacteriologic surveillance showed the rapid clonal spread of non-serogroup B of meningococcus especially in the special population such as college students or military personnel, the policy of public meningococcal vaccination should be reevaluated.

In the Saudi Arabian study by Lingappa JR et al, comparing patients with the epidemic strain of serogroup W135 and those with serogroup A [3], the former were less likely to have meningitis than the latter [3]. Similarly, we found a statistically nonsignificant trend toward more pure bacteremia without meningitis among patients with sporadic W135 disease, compared to patients with non-W135 disease. Our finding that more pneumonia and less CSF inflammatory cells in patients with W135 meningitis than in patients with non-W135 meningitis was similar to findings in France that the predominant extrameningeal diseases (such as pneumonia, septic meningococcal arthritis and pericarditis) occurred predominantly in patients with W135 disease [10-13]. However, the use of a different definition, and failure to use PCR as diagnostic tool may have resulted in less association between arthritis and W135 in our series.

Similar to previous reports [22-24], we found that pure bacteremia without meningitis, altered mental status, peripheral white cell counts less than 10,000/mm^3^, thrombocytopenia < 100,000/mm^3^, initial systolic blood pressure < 85 mmHg, and purpura or petechiae on admission were factors associated with mortality in patients with meningococcal disease. There are several scoring systems developed for assessing the prognosis and mortality of meningococcal disease [22-25], Glasgow Meningococcal Septicemia Prognostic Score is a good example of a clinical prediction tool for assessing the mortality of meningococcal disease [22,23]. Most of those scoring systems are based on easily available clinical and laboratory parameters such as differences in age distribution, period between onset of disease and admission, absence of meningitis, presence of widespread skin lesions, hypotension, metabolic acidosis, normal C-reactive protein level, absence of leukocytosis, presence of thrombocytopenia, and hypofibrinogenemia [22-25]. However, some of the above factors (e.g., C-reactive protein and fibrinogen levels) were not routinely checked in our cases.

Previous studies that included only a low percentage of patients with serogroup W135 meningococcal disease could not comment on the influence of W135 on outcome [23-25]. Our present series, with a higher percentage of patients with serogroup W135 disease, showed no difference in case-fatality rates between patients with W135 and patients with non-W135. Lingappa JR et al [3] also found no difference in the case fatality rate between patients with serogroup W135 and serogroup A in Saudi Arabia, but more patients with W135 needed intensive care. There was a statistically insignificant trend toward more shock and intubation in patients with W135 compared with those with non-W135 in our series.

## Conclusion

In conclusion, sporadic W135 infections accounted for a substantial proportion of meningococcal disease in Taiwan from 2001 through 2003 and do not appear to be Hajj-related. More patients age 20–54 years, and not younger, developed the disease in Taiwan. The clinical features of W135 and non-W135 meningococcal disease were similar except that patients with W135 disease were more likely to have extrameningeal involvement such as pneumonia. Independent predictors of mortality included bacteremia without meningitis, altered mental status, and petechiae or purpura on admission.

## Competing interests

The author(s) declare that they have no competing interests.

## Authors' contributions

Wang JL and Liu DP participated in the design of the study and data analysis. Yu CJ and Liu HC carried out the data collection. Lin CY participated in the study of complements. Chang SC and Yen JJ conceived of the study, and participated in its design and coordination and helped to draft the manuscript. All authors read and approved the final manuscript.

## Pre-publication history

The pre-publication history for this paper can be accessed here:


